# Laparoscopic ureterolithotomy for lower ureteric stones: Steps to make it a simple procedure

**DOI:** 10.4103/0970-1591.45556

**Published:** 2009

**Authors:** Anil Mandhani, Rakesh Kapoor

**Affiliations:** Department of Urology, Sanjay Gandhi Post Graduate Institute of Medical Sciences, Lucknow, Uttar Pradesh-226 014, India

**Keywords:** Laparoscopy, lower ureteric stone, ureterolithotomy

## Abstract

Despite advances in endoscopy and availability of holmium lithotripsy there are ureteric stones, which primarily need to be treated with laparoscopic ureterolithotomy. Literature is replete with the stone retrieval in upper ureteric stone but there are a very few reports on stones removal from ureter below the lower sacroiliac joint. Putting a double J stent before starting the procedure does not give any extra advantage; rather it takes away significant operating room time. This point of technique describes port placement strategy, proximal ureteral occlusion; stone localization, ureterotomy, stone retrieval and laparoscopic stenting are the important steps where one would like to be careful enough to complete the procedure successfully.

## INTRODUCTION

Laparoscopic ureterolithotomy is an alternative to open surgery for removing large stones not amenable to endoscopic treatment.[1,2] In most of the published literature, laparoscopic approach for lower ureteric stone is described to be less successful than middle and upper ureter.[3,4] Upper and mid ureteric stones are safely approached retroperitoneally but lower ureteric stones are better approached transperitoneally.[5] This article describes important technical points to successfully retrieve large lower ureteric stones through transperitoneal laparoscopy.

## TECHNIQUE

**Figure 1:**
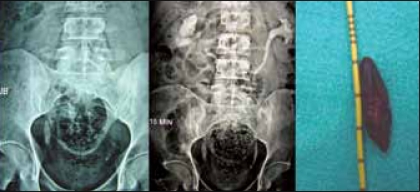
Port placement strategy based on the surface marking of the stone

Two patients with lower ureteric stone of size of 3 cm and 2.8 cm were primarily treated with laparoscopy. Both the affected units had good renal function on intravenous urogram [Figure 1].

### Prior placement of the double J stent

Placement of double J stent prior to laparoscopy is not only fraught with difficulty but also takes away precious time of the operating room. It could safely be avoided since it does not help in either localization of the ureter or stone during laparoscopy. With the technique described subsequently, it takes very little time to place the double J stent safely once the stone is taken out from the ureter.

### Port placement strategy

**Figure 2:**
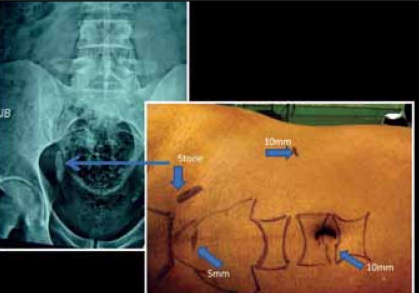
A 3 cm stone in the lower ureter

Patient was placed in 45° lateral position with the operating side up. Location of the stone on the body surface in relation with bony landmarks was marked to help placing the ports. Camera port was placed at the umbilicus with open technique. Dominant port of 11 mm was inserted under vision in the iliac fossa [Figure 2] and the non dominant port of 5 mm at the suprapubic area.

### Mobilization of the colon and reaching the ureter

**Figure 3:**
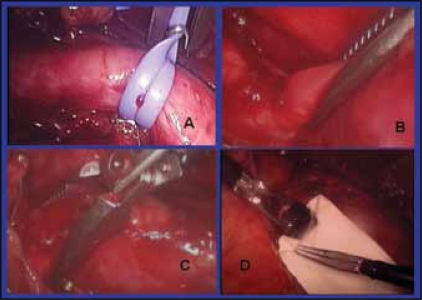
Important surgical steps e.g., A proximal control of the ureter, B and C ureteral pinching and D bagging the stone

As soon as colon was reflected, iliac vessels were identified and then it became easy to identify the ureter, which was then circumscribed with the vascular loop [Figure 3a]. Ureter was then dissected distally staying away from the adventitia till the stone site was reached. Superior vesical artery was also encountered in both the cases, which could safely be sacrificed.

### Localization of the stone by “Ureteral pinching”

Due to absence of haptic feedback, exact site of incision over the stone sometimes becomes challenging. Pinching the ureter gently gave us the exact location of the stone. Using Maryland dissector, a non stone bearing part of the ureter could be pinched fully [Figure 3B], but the stone carrying part could not [Figure 3C].

### Ureterotomy and trapping the stone

Once the stone was localized by ‘ureteral pinching’, pointed diathermy hook was used to incise the ureter over the stone. Maryland dissector was used to fish out the stone with closed forceps’ tip or using its one prong only. The same dissector could be used to hold the stone and bag it in the glove finger, which was then attached with a clip to the parietal wall for its removal at the end of surgery [Figure 3D].

### Laparoscopic stenting

#### Stent assembly

**Figure 4:**
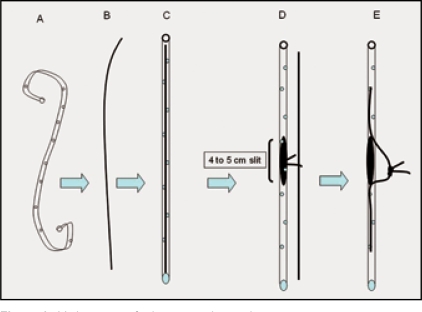
Various steps for laparoscopic stenting

For making a safe placement of the stent and to obviate the need for fluoroscopic confirmation, mathematical calculation helped to achieve the goal of placing the stent correctly. Bony landmark for the ureteric orifice is 1 cm above and lateral to the pubic tubercle. Exact distance from this point to the stone on an X-ray KUB was measured. This corresponded with the site for making a slit in the double J stent with 11 number surgical blade. Closed end of the stent was left as such and a guide wire cut a little short of the length of the stent was inserted to make both the ends of the double J stent straight [Figure 4]. A prolene thread was tied to the guide wire at the slit with multiple knots. This helped in pulling the guide wire out once the stent was positioned.

### Placement of the double J stent

Once the stone was taken out, one end (usually the closed end) of the stent was put in proximally to its complete length. As proximal ureter is dilated, pushing the whole length of the stent with knot on it was not a problem. Then, holding the other end of the double J stent (which was straight due to presence of a guide wire); stent was pushed in distally till the knot was visualized at the ureterotomy site. Supporting the stent with Maryland in left hand and holding the knot with right hand instrument, guide wire was taken out. This ensured safe placement of the double J stent [Figure 4].

### Suturing the Ureterotomy incision

Once the stent was in place, 4-0 vicryl was used to close the ureterotomy with interrupted stitches and a tube drain was placed before closing the ports. Operating time in both the cases was 120 min and 130 min, respectively. Urethral catheter was taken out first and then the drain, when drainage was less than 30 cc. Two cases done so far did not have any intraoperative or postoperative complications. Double J stent was taken out after 6 weeks and at the mean follow up (follow up was done with assessment of clinical symptoms and ultrasonography) of 5 months both the patients are doing well.

## DISCUSSION

Laparoscopic ureterolithotomy is a minimally invasive option to treat large ureteric stones not amenable to ureteroscopy. There are a very few small case series describing laparoscopic ureterolithotomy for lower ureteric stone.[4,5] Though the mid and upper ureteric stones which are too large to be retrieved ureteroscopically, are safe to remove retroperitoneally, lower ureteric stones are little demanding due to its complex location in the pelvis. In one study, iliac retroperitoneal approach has been described for stones below ischial spine. In that study, reflection of peritoneum over the iliac vessels and inflamed ureter below the sacroiliac joint was found to be difficult so there were only 2 cases done out of total 101 patients.[6] Transperitoneal approach gives better understanding of the anatomical landmarks particularly for the lower ureteric stone.

Port placement is the most crucial part of any laparoscopic surgery, more so for approaching lower ureteric stones located below the Sacroiliac joint (SI). As per the protocol, we at our institute adopted an open technique for placement of the camera port in all our laparoscopic surgeries. It is easy to enter the abdomen at the umbilicus as only rectus sheath is encountered. Most of the laparoscopic surgeon, wittingly or unwittingly follow a strategy of diamond shape configuration for port placement where the target area lies in the center. Surface marking for lower ureteric stone helped in exact placement of dominant and assistant ports.

It is always better to dissect the ureter proximal to the site of the stone as it is free from periureteral inflammation and fibrosis. Most of the time, the stone is impacted due to surrounding inflammation and does not move with manipulation but secondary stones proximal to it may move proximally due to manipulation of the ureter. Therefore, as soon as ureter is identified at the iliac vessels it should be double looped with vascular tape and then dissection should be carried out distally.

Stone localization is an important step of this surgery. In case, when the ureter is not so dilated, stone could be seen bulging in the ureter but in cases where stone is not seen prominently due to proximal dilatation of the ureter, it would sometimes become difficult to localize the stone visually. Pinching with the Maryland forceps helps in localizing the stone. Fluoroscopic localization would help if one is not sure about it but in both these cases stone could be localized by pinching technique. Similarly while making ureterotomy with electrocautery; it did not require fixing of stone in one position as done while using cold knife and it also gave better hemostasis. It has been shown that using diathermy to make ureteral incision does not affect the ureteric tissue healing adversely.[6]

Once the stone is fished out, it should be bagged. In retroperitoneal surgery for upper ureteric stones, where cup forceps have been described to pull the stone along with the port, wherein even if a stone slips out then due to limited space in the retro peritoneum it could be retrieved again.[7] Unlike in retroperitoneal approach it is always better to bag the stone to avoid the risk of losing it in the peritoneal cavity.

In large stones with presence of inflammation it is always better to place a double J stent that is put in before or during the laparoscopy to avoid the complication of urinary extravasation and urinoma formation.[4] Many a times due to impaction and inflammation, it is not possible to place a double J stent cystoscopically despite using various manoeuvres and ureteroscopic guidance. If double J stent could be placed laparoscopically after the removal of stone then it not only saves significant anaesthesia time but also operating room resources. Laparoscopic stenting after ureterolithotomy has been described with a custom made stent where both the ends are closed with a guide wire protruding out in the middle forming a loop.[8] Disadvantage of this is that the site of loop does not correspond to the site of the stone and stent requires to be manufactured as an additional thing whereas in current technique the routinely available stent could be used. In both the cases stent could be placed safely.

## CONCLUSIONS

With modified technique of laparoscopic stenting and ureteral pinching and port placement strategy, lower ureteric stones could be well treated with transperitoneal laparoscopic approach.
